# Development and Preliminary Validation of the Divine Connectedness Scale in the USA

**DOI:** 10.1007/s10943-024-02111-7

**Published:** 2024-08-25

**Authors:** Jesus Alfonso D. Datu, Frank D. Fincham

**Affiliations:** 1 Centre for Advancement in Inclusive and Special Education, Academic Unit of Human Communication, Learning, and Development, The University of Hong Kong, Rm. 210 Runme Shaw Bldg., Pokfulam Rd., Hong Kong, Hong Kong SAR China; 2 Family Institute, Florida State University, Tallahassee, FL USA

**Keywords:** Divine relatedness, Subjective well-being, Flourishing, Religiosity

## Abstract

This research conceptualized and offered preliminary evidence on the psychometric properties of the 10-item Divine Connectedness Scale—a measure that assesses individuals’ perceptions of feeling supported by (*divine guidance*) and working with (*divine collaboration*) God or a Supreme Being. Results of exploratory factor analysis and confirmatory factor analysis with 434 undergraduate students in the United States showed that scores from a single-factor model of divine connectedness were valid and reliable. Divine connectedness was positively associated with religiosity, forgiveness, and well-being variables. Divine connectedness showed incremental validity over demographic covariates, social desirability, and religiosity in predicting later meaning in life and flourishing.

## Introduction

Psychologists have long recognized the benefits of feeling connected to human beings and broader social contexts (Baumeister & Leary, 1995). For instance, studies have shown that students with greater perceptions of connectedness to parents (Datu & Yuen, 2020; Furrer & Skinner, 2003), peers (Jia et al., 2009), and teachers (Paniagua et al., 2022) tend to have greater well-being and positive academic outcomes (e.g., academic achievement and engagement). In addition, individuals with greater perceived belongingness to specific contexts such as schools (Gale & Nepomnyaschy, 2023; Marraccini & Brier, 2017), identity-specific communities (e.g., transgender, and nonbinary individuals’ community; Taber et al., 2023), and nature (Pritchard et al., 2020) reported greater well-being.

Investigating people’s sense of connectedness to religious organizations or religion in general, is an essential research initiative for a few reasons. In 2021, about three-in-ten or 30% of Americans did not identify themselves with any religion (Pew Research Center, 2021). Although it was estimated in 2020 that 64% of the United States population are Christians, this number is expected to shrink by 2070 to as low as 35% (Pew Research Center, 2022). As these statistics project significant secularization trends in the U.S. and other cultural contexts, scientific questions that revolve around specific reasons accounting for the sharp decline in religious affiliations are of utmost importance.

Against this backdrop, the present research conceptualizes and provides preliminary evidence on the psychometric validity of the Divine Connectedness Scale (DCS) among undergraduate students in the United States. We also explore the criterion-related validity of the DCS by assessing its correlations with religiosity (Fincham & May, 2021), dispositional tendency to forgive one’s self, others, and situations (Thompson et al., 2005), subjective well-being dimensions such as life satisfaction, positive emotions, and negative emotions (Diener et al., 1985), presence of meaning in life (Steger et al., 2006), and psychological flourishing or perceived social-psychological prosperity as characterized by self-efficacy, meaning, and positive relationships (Diener et al., 2009). To generate insights into the Divine Connectedness Scale’s discriminant validity, we explore whether it relates to the expression of gratitude to others (Lambert et al., 2010) and social desirability (Hart et al., 2015). Further, we examine whether divine connectedness shows incremental validity over age, gender, social desirability, and religiosity in predicting well-being outcomes after 8 weeks.

### Relationship to God and Theoretically Related Constructs

Early models of connectedness to religious organizations tended to focus on the motives and orientation of religious activities. For instance, Gorsuch and McPherson (1989) created the Intrinsic/Extrinsic Religious Connectedness Scale to measure personal and social reasons for performing religious or Church-specific behaviors. Adaptations of these scales—by replacing items referring to Christian beliefs with other religious affiliations, such as Islamic ones for Muslim people—resulted in good psychometric properties (Ali Alzahrani & Sehlo, 2013; Bouchard et al., 1999). In addition, Rogers-Dulan (1998) developed and validated the Religious Connectedness Questionnaire which assesses individuals’ perceived engagement in the context of personal, familial, and Church-specific religious activities. Importantly, people with greater perceived religious connectedness based on such measures reported optimal psychological functioning (Ali Alzahrani et al., 2013; Rogers-Dulan, 1998). Although both scales can provide an estimate of the extent to which individuals engage in intrinsically or extrinsically driven religious activities, such conceptual and measurement models do not offer more precise insights into people’s sense of connection to the Supreme Being in specific religious affiliations (e.g., God for Christians and Allah for Muslims).

Other empirical investigations have explored religious psychological constructs that may resemble connectedness to God. For instance, adolescents who have a greater perceived relationship with God tend to have lower internalizing symptoms (Goeke-Morey et al., 2014). Female undergraduate students with stronger perceptions of relationship to God—measured by the Attachment to God Inventory (Beck & McDonald, 2004)—tend to have greater well-being (Homan & Cavanaugh, 2013).

Further, some studies have indicated how God-mediated control beliefs—encompassing one’s capacity to collaboratively work with God or the Supreme Being to chart a course of actions or solutions to problems in their lives (Krause, 2005)—matter for a few key physical and psychological outcomes. For instance, people with greater God-mediated control beliefs reported greater perceived meaning in life and optimism (Krause, 2010) and life satisfaction (Krause, 2005). Evidence also shows that God-mediated control beliefs buffered the negative impacts of stressful life events on well-being outcomes (Krause, 2019). Indeed, these findings suggest that people’s perceptions of mutually cooperating with God in shaping life events have implications for understanding connectedness to God.

More recently, Hall et al. (2022) have conceptualized the intimacy with God construct—a term that characterizes Christians’ perceptions of intimate ties to God. They built a three-dimensional model of intimacy with God—*mutual presence, mutual closeness,* and *intersubjectivity*–based on prior literature about the psychological foundations of social intimacy. First, *mutual presence* involves being aware that God is present. Second, *mutual closeness* pertains to the tendency to reveal oneself to God and actively respond to God’s revelation. Third, *intersubjectivity* refers to perceived oneness in terms of God’s mind and motivation (e.g., believing in the value of greater commitment to God translates to a stronger commitment to a relationship with intimate partners). The findings of this study, however, demonstrated that there was no evidence to clearly differentiate such dimensions of intimacy with God; thus, a single factor is recommended in using the Intimacy with God Scale.

### Divine Connectedness

In this research, we extend existing scientific debates on connectedness in specific domains of relationships that have typically focused on connectedness to intimate partners, social ties (e.g., friends), and community (Baumeister & Leary, 1995; Stavrova & Luhmann, 2016) by conceptualizing divine connectedness—a construct that encompasses the extent to which individuals feel that they are connected to God. Divine connectedness is hypothesized to involve two components: (1) *divine guidance*, which refers to a sense that one is guided or supported by God, and (2) divine collaboration, which pertains to the perception that one is collaborating with God in shaping important events or outcomes of various actions in their lives. Our conceptualization resonates with previously identified dimensions of connectedness in specific domains such as perceived support from a specific social agent or environmental context (Goodenow, 1993; Perkins et al., 2021) and active engagement in collaborative activities in specific contexts (Hare-Duke et al., 2019; Krause, 2005).

Specifically, this study develops and provides preliminary evidence on the psychometric properties of the Divine Connectedness Scale—a measure that assesses the extent to which one experiences divine guidance and divine collaboration—among undergraduate students in the United States. As with prior recommended guidelines in psychological scale construction (Porter & Fabrigar, 2007), we randomly divided our sample into exploratory and cross-validation samples. In the exploratory sample, we conducted an exploratory factor analysis to identify the optimal factor solution that characterizes the divine connectedness construct. In the cross-validation sample, the results of the exploratory factor analysis were explored using confirmatory factor analysis.

Based on related prior research findings, we hypothesize that divine connectedness will positively correlate with concurrent and subsequent religiosity, dispositional forgiveness, positive emotions, life satisfaction, meaning in life, and flourishing. However, we anticipated that divine connectedness would be related to lower negative emotions.

## Methods

### Participants and Procedures

Participants (*n* = 434) were recruited from undergraduate courses at a public university in the southeast of the United States. The vast majority were from the human and social sciences, and most students in these departments and colleges were female. They were offered options to earn a small amount of extra credit for their course, one of which was completing online surveys that included the questionnaires reported in this study The participants’ average age was 19.91 with a standard deviation of 1.82. The majority of participants were female (*n* = 392). Most of them reported that they were Christians (*n* = 347).

Prior to data collection, the first author sought approval to conduct the survey from the institutional review board at the Florida State University. Then, online surveys containing consent forms were distributed to target participants at two time points separated by 8 weeks. This study was part of a project that identified longitudinal outcomes of religious behaviors and optimal psychological functioning in the United States.

### Measures

#### Divine Connectedness

We developed the 11-item Divine Connectedness Scale (CDS) to assess the perceived sense of connection to God, characterized by divine guidance and divine collaboration. In constructing this measure, we followed existing methodological guidelines in creating new psychological scales (DeVellis, 2012; Koenig & Al Zaben, 2021) which involved operationalizing domains or dimensions of divine connectedness with reference to prior studies (Krause, 2005; Perkins et al., 2021; Rogers-Dulan, 1998), writing items, administering the scale, conducting factor analytic tests, and examining evidence of criterion-related, convergent, discriminant, and incremental validity. Items were rated on a 5-point Likert scale (1 = Strongly disagree; 5 = Strongly agree). Cronbach’s alpha coefficients of this scale at Time 1 and Time 2 were 0.97 and 0.96.

#### Expression of Gratitude Scale to Others

We used the 3-item modified version of the Expression of Gratitude in Relationships Scale (Lambert et al., 2010) to assess the participants’ reports of expressing appreciation to other people. Items were rated using a 5-point response option (1 = Never; 5 = Very frequently). Sample items in the scale include: “I express my appreciation for the things that other people do for me.” Cronbach’s alpha coefficient of this scale in this study was 0.74.

#### Forgiveness

The 18-item Heartland Forgiveness Scale (Thompson et al., 2005) assessed participants’ perceived tendency to forgive themselves, others, and situations. Items were rated using a 7-point response scale (1 = Almost always false of me; 7 = Almost always true of me). Sample items include: “Although I feel badly at first when I mess up, over time I can give myself some slack.” (forgiveness of self), “With time I am understanding of others for the mistakes they’ve made.” (forgiveness of others), and “I eventually make peace with bad situations in my life.” (forgiveness of situations). In this study, Cronbach’s alpha coefficients of the forgiveness of self, others, and situations subscales were 0.76, 0.82, and 0.78, respectively.

#### Religiosity

Two items commonly used to assess religiosity in past research (Fincham & May, 2021) were used in this study. The two items were: “How committed are you to your beliefs about religion?” and “How often do you attend religious/spiritual services or meetings?” Items were rated on an 8-point scale (1 = Not very committed; 8 = Extremely committed). Cronbach’s alpha coefficient of this scale in this study was 0.73.

#### Positive and Negative Emotions

The 12-item Scale of Positive and Negative Experience (Diener et al., 2009) was used to assess the extent to which the respondent experienced positive and negative emotions in the past four weeks. Items were rated using a 5-point Likert scale (1 = Very rarely or Never; 7 = Very often or Always). Here are sample items: a) positive emotions = “positive” and “good”; and b) negative emotions = “negative” and “bad.” Cronbach’s alpha coefficients of the positive emotions scale at Time 1 and Time 2 in this study were 0.83 and 0.80. Further, Cronbach’s alpha coefficients of the negative emotions scale at Time 1 and Time 2 in this study were 0.85 and 0.88.

#### Satisfaction with Life

The 5-item Satisfaction with Life Scale (Diener et al., 1985) assessed perceived contentment with life. Items were rated using a 7-point Likert scale (1 = Strongly disagree; 7 = Strongly agree). Sample items in the scale include: “In most ways, my life is close to ideal.” Cronbach’s alpha coefficients of this scale at Time 1 and Time 2 in this study were 0.90 and 0.91, respectively.

#### Social Desirability

We used the 8-item impression management subscale in the Balanced Inventory of Desirable Responding Short Form (Hart et al., 2015) to assess the role of socially desirable responses in this study. This subscale comprises items that capture “a conscious dissimulation of responses to create a socially desirable image” (Hart et al., 2015, p. 2) and validity data include a substantial correlation with the longer Marlowe-Crowne Social Desirability Scale (*r* = 0.53). The items were rated on a 7-point Likert scale (1 = Strongly disagree; 7 = Strongly agree). Sample item includes: “I sometimes tell lies if I have to.” Cronbach’s alpha coefficient of this scale in this study was 0.66.

#### Meaning in Life

We used the 5-item presence of meaning in life subscale of the Meaning in Life Questionnaire (Steger et al., 2006) to assess participants’ perceptions of the degree to which they understood and appreciated the meaning of their existence. Items were rated on a 7-point scale (1 = Absolutely untrue; 7 = Absolutely true). Sample items include: “I understand my life’s meaning.” In this study, Cronbach’s alpha coefficients of this scale at Time 1 and Time 2 were 0.89 and 0.89.

#### Psychological Flourishing

The 8-item Psychological Flourishing Scale (Diener et al., 2009) was used to assess social psychological prosperity as evidenced by engagement, optimism, purpose in life, and positive relationships. Items were rated using a 7-point Likert scale (1 = Strongly disagree; 7 = Strongly agree). Sample items used in this scale include: “I am engaged and interested in my daily activities” and “My social relationships are supportive and rewarding.” Cronbach’s alpha coefficients of this scale at Time 1 and Time 2 in this study were 0.96 and 0.97.

### Data Analyses

Before the main data analyses, we used the SPSS 28v to randomly divide our total sample into two subsets (i.e., the first subset had 207 students and the second subset had 227). In the exploratory sample, we conducted principal component analysis via direct oblimin rotation using SPSS 29v to identify the ideal factor solution of the newly developed DCS. As with prior methodological guidelines, we retained items with factor loadings that exceed 0.50 and maintained factors with at least three items (DeVellis, 2012).

In the cross-validation sample, we performed a confirmatory factor analysis (CFA) via maximum likelihood estimation using JASP 0.17.2.1 to assess the fit of the optimal factor structure of divine connectedness. In evaluating the fit of the proposed measurement model, we used the following guidelines: a) Comparative fit index (CFI) that should be greater than 0.90, and b) root mean square error of approximation (RMSEA) and standardized root mean square residual (SRMR) that should be lower than 0.08.

Cronbach’s alpha coefficients and descriptive statistics (i.e., mean, and standard deviation values) of the divine connectedness scale and other criterion measures were calculated. To examine the test–retest reliability of this scale, we conducted Pearson-r correlational analyses between the composite score on divine connectedness at Time 1 and Time 2 (administered 8 weeks after the first phase of data collection). To assess the convergent and discriminant validity of this scale, we conducted correlational analyses to examine the associations of divine connectedness with religiosity, expression of gratitude to others, dispositional forgiveness dimensions, and well-being criterion variables (i.e., life satisfaction, positive emotions, negative emotions, presence of meaning in life, and flourishing). To generate insights about the scale’s incremental validity, we performed hierarchical regression analyses—which involved adding age, gender, social desirability, and religiosity as Step 1 predictors and Time 1 divine connectedness as a Step 2 predictor—using the dataset with completed responses (n = 135) to assess the links of Time 1 divine connectedness to Time 2 life satisfaction, positive emotions, and negative emotions when controlling for demographic covariates, social desirability, and religiosity. Descriptive, reliability and correlational analyses were conducted using the SPSS 29v.

## Results

Preliminary analyses indicate that the dataset in the exploratory sample was suitable for factor analysis. The Kaiser–Mayer–Olkin (KMO) index was 0.945 and Barlett’s test of sphericity was statistically significant (*χ*^*2*^ = 2416.42, *p* < 0.001), suggesting that factor analysis is appropriate in our study based on conventional guidelines (DeVellis, 2012). The results of principal component analysis via promax rotation showed that two factors exceeded an Eigenvalue of 1, which accounted for 70.35% and 9.69% of the variance in divine connectedness, respectively. However, a review of the rotated component matrix demonstrated that all items except the reverse-worded item no. 6 (“You decide what to do without relying on God.”) loaded onto the first factor. Factor loadings of the 10 items that loaded on the first factor ranged from 0.68 to 0.93 (see Table 1 to review the factor loadings of all items). Hence, we disregarded the second factor as only the reverse-worded item loaded on that factor. The Cronbach’s alpha coefficient of the final 10-item DCS was 0.97.

**Table 1:** Factor loadings of DCS’ items based on the rotated component matrix of principal component analysis via direct oblimin estimation rotation approach

Items	Factor 1	Factor 2
8. I recognize how God guides me in coping with various challenges in life	.92	
11. I work with God to overcome difficulties related to achieving my personal ambitions	.92	
9. I feel that God pushes me to stay strong in hard times	.91	
10. I recognize God’s active role in facilitating positive changes in my life	.90	
3. I believe that God constantly assists me in doing daily activities	.90	
5. With God’s guidance, I am able to set realistic ambitions for myself	.89	
4. I consult God when making major life decisions	.88	
2. I feel that ‘working with’ God enables me to achieve various goals in life	.87	
7. Nothing is impossible if I work together with God	.85	
1. I feel that God supports me in everything that I do	.71	
6. You decide what to do without relying on God		.95

In the cross-validation sample, the results of confirmatory factor analysis via maximum likelihood estimation using JASP 0.17.2.1 showed that the single-factor model of the divine connectedness construct had an acceptable fit except for the RMSEA: *χ*^*2*^ = 138.06, *df* = 35; *CFI* = 0.97, *RMSEA (90% C.I.)* = 0.114 (0.094, 0.134); *SRMR* = 0.02. All item indicators significantly loaded onto the hypothesized single-factor divine connectedness latent construct. We tested a two-factor model of divine connectedness with divine guidance and divine collaboration as correlated factors, but it did not converge. Thus, we used the single-factor model of divine connectedness in the succeeding analyses. The divine connectedness scale had a high internal consistency coefficient (*α* = 0.97). The test–retest correlational coefficient between Time 1 and Time divine connectedness was acceptable (*r* = 0.79). Table 2 reports the findings of descriptive statistical analyses.

**Table 2:** Cronbach’s alpha coefficients and descriptive statistics of divine connectedness and criterion measures

	***α***	***M***	***SD***
1. T1 Divine connectedness	.97	3.29	1.09
2. T1 Social desirability	.67	4.05	0.85
3. T1 Religiosity	.73	5.18	1.95
4. T1 Expression of gratitude to others	.74	4.60	0.46
5. T1 Positive emotions	.83	3.37	0.57
6. T1 Negative emotions	.85	2.40	0.70
7. T1 Life satisfaction	.90	5.26	1.27
8. T1 Presence of meaning	.89	5.33	1.18
9. T1 Flourishing	.96	5.68	1.12
10. T1 Forgiveness of self	.76	4.75	0.96
11. Forgiveness of others	.82	4.91	1.06
12. Forgiveness of situations	.78	4.78	1.04
13. T2 Positive emotions	.80	3.40	0.55
14. T2 Negative emotions	.88	2.39	0.72
15. T2 Life satisfaction	.91	5.57	1.12
16. T2 Presence of meaning	.89	5.49	1.11
17. T2 Flourishing	.97	5.90	1.08

Results of correlational analyses showed divine connectedness was significantly correlated with theoretically related criterion variables in the cross-validation sample (see Table 3). For instance, divine connectedness had strong positive correlations with religiosity. Divine connectedness was also positively associated with Time 1 life satisfaction, Time 1 positive emotions, Time 1 presence of meaning, and Time 1 psychological flourishing. In addition, divine connectedness is positively correlated with forgiving others and situations. However, divine connectedness did not have significant associations with expressing gratitude to others and self-forgiveness. Further, divine connectedness was positively correlated with Time 2 positive emotions, Time 2 presence of meaning in life, and Time 2 flourishing. Divine connectedness had negative correlations with Time 1 and Time 2 negative emotions. Table 4 reports the findings of correlational analyses between Time 1 divine connectedness and Time 2 well-being criterion variables.

**Table 3:** Correlations of divine connectedness with concurrent criterion-related validity measures

	T1 religiosity	T1 impression management	T1 positive emotions	T1 negative emotions	T1 life satisfaction	T1 presence of meaning	T1 flourishing	T1 expression of gratitude to others	T1 forgiveness of self	T1 forgiveness of others	T1 forgiveness of situations
Divine connectedness	.70***	.13*	.15*	−.16*	.19**	.26***	.20**	.06	.12	.23***	.19**

***p < .001, **p < *.01, p* < *.05*

**Table 4:** Correlations of divine connectedness with subsequent well-being criterion variables

	T2 positive emotions	T2 negative emotions	T2 life satisfaction	T2 presence of meaning	T2 flourishing
T1 divine connectedness	.18*	-.23**	.16	.33***	.29***

***p < .001, **p < *.01, p* < *.05*

We conducted four separate regression analyses to assess whether Time 1 divine connectedness predicted Time 2 life satisfaction, Time 2 positive emotions, Time 2 negative emotions, and Time 2 flourishing when controlling for the effects of age, gender, social desirability, and religiosity. The regression model for Time 2 flourishing was significant, *R*^*2*^ = *0.12, F(*45 127*)* = 3.360 *p* < *.* 05. Time 1 divine connectedness accounted for variance in this criterion variable above and beyond the effects of demographic covariates, socially desirable responding, and religiosity, *ΔR* = 0.03, *β* = 0.25, *t* = 2.04, *p* < 0.05. Similarly, The regression model for Time 2 presence of meaning in life was significant, *R*^*2*^ = *0.13, F(*5, 127*)* = 3.91, *p* < *.* 001. Time 1 divine connectedness accounted for additional variance in the presence of meaning in life above and beyond the effects of demographic covariates, socially desirable responding, and religiosity, *ΔR* = 0.04, *β* = 0.29, *t* = 2.28, *p* < 0.05. Although the regression model for Time 2 positive emotions was significant, *R*^*2*^ = *0.*11, *F*(5, 127) = 3.05, *p* < *.* 05), divine connectedness did not predict this criterion variable. Further, the regression models for Time 2 life satisfaction (*R*^*2*^ = 0.07, *F(*5, 127*)* = 1.95, *p* = *.* 09) and negative emotions were not significant, *R*^*2*^ = 0.07,* F*(5, 127) = 1.80, *p* = 0.12.

## Discussion and Conclusions

Connectedness to different social agents and even non-social entities (e.g., nature and neighborhood) has gained traction in the extant psychological literature. However, scientific research on what constitutes connectedness to God or a Supreme Being remains elusive. The current research addresses this gap by developing and preliminarily validating the Divine Connectedness Scale—a measure that assesses one’s sense of connectedness to God involving the subjective experience of receiving guidance and support from God and collaborating with God in shaping key life events and personal decisions.

Results of exploratory and confirmatory factor analyses showed that the unidimensional model of the divine connectedness construct had the most optimal fit among undergraduate students in the United States. This scale had high internal consistency and test–retest reliability coefficients. Although we originally conceptualized divine connectedness as tapping into two dimensions—namely *divine guidance* and *divine collaboration*—of individuals’ perceived connection to God or a Supreme Being, findings of factor analytic approaches did not corroborate this prediction. That *divine guidance* and *divine collaboration* did not emerge as separate dimensions of the divine connectedness construct indicates that both components are deeply intertwined and concurrently experienced when operationalizing one’s close relationship with God. Our study coheres with prior research that showed a lack of differentiation in the specific facets or dimensions of religious psychological constructs (Hall et al., 2022).

Consistent with our predictions, the data in our study showed that divine connectedness was associated with higher concurrent religiosity, forgiveness of others and situations, life satisfaction, positive emotions, and psychological flourishing. Further, divine connectedness was linked to higher positive emotions and flourishing after 8 weeks. Conversely, divine connectedness was linked to lower concurrent and subsequent negative emotions. In addition, divine forgiveness’s weak to non-significant correlations with theoretically distinct constructs such as social desirability and expression of gratitude to others provide preliminary evidence of the DCS’ discriminant validity. Importantly, divine connectedness uniquely accounted for variance in subsequent meaning in life and flourishing, even when controlling for the effects of age, gender, social desirability, and religiosity. This clearly demonstrates that divine connectedness did not simply serve as a proxy measure of religiosity. It is plausible that connectedness to God may be linked to greater well-being by promoting a sense of purpose in life (Krause, 2004) and self-esteem (Krause, 2005)—conditions that can foster subjective and psychological well-being. In general, these results align with past studies that show people with better perceptions of their relationship with God tend to report higher perceived physical and psychological well-being outcomes (Homan & Cavanaugh, 2013; Krause, 2005, 2010; Hall et al., 2022).

Further, our results indicate that divine connectedness was more strongly associated with criterion measures or variables (i.e., meaning in life and flourishing) tapping features of eudaimonic well-being (Ryff, 1989). Divine connectedness may relate to dimensions of eudaimonic well-being if it contributes to psychological processes (e.g., effective emotion regulation strategies and confidence in maintaining relationships) that can result in self-growth and social well-being. In general, these findings cohere with past studies that emphasized the critical role of connectedness to specific social agents or other related domains (Baumeister & Leary, 1995; Ryan & Deci, 2001) in facilitating optimal psychological functioning.

### Limitations and Future Research Directions

Our findings should be interpreted in light of the following shortcomings. Given the preliminary nature of this scale construction and poor RMSEA estimate in the measurement model, findings cannot generate insights into the robustness of the unidimensional factor structure of divine connectedness construct in other undergraduate student and adult samples in the United States. Future investigations might explore the psychometric validity of the Divine Connectedness Scale using alternative statistical modeling approaches (e.g., item response theory analysis). Our reliance on self-report measures of divine connectedness and other criterion measures might have increased common method bias in this study. Future studies may adopt alternative data collection approaches to assess subjective and psychological well-being (e.g., physiological markers of positive emotions) to provide more rigorous evidence on divine connectedness’ links to optimal psychological functioning. In addition, our focus on measuring divine connectedness in the United States has corresponding constraints on the generalizability of our findings to non-Western cultural contexts. Assessing the cultural applicability of the divine connectedness construct in non-Western and collectivist societies is an important scholarly direction that will enrich our insights into the cultural relevance and psychological benefits of being connected to God in diverse cultural contexts.

## Conclusion

Our study contributes to the existing literature by conceptualizing divine connectedness—a construct that encompasses a perceived sense of being guided and supported by God and having collaborative ties with God in shaping events and important actions or decisions in life. Importantly, we demonstrated that the newly developed Divine Connectedness Scale had acceptable psychometric properties in a sample of undergraduate students in the United States. Thus, future research can use this assessment tool to measure people’s perceptions of their connectedness to God. Further, our results on the links of divine connectedness to concurrent religiosity, life satisfaction, positive emotions, presence of meaning in life, and flourishing clearly indicate that this scale showed good criterion-related validity. Importantly, this study demonstrates that divine connectedness uniquely contributes to variance in meaning in life and psychological flourishing after 8 weeks, even after accounting for the effects of religiosity. We hope this research can stimulate ongoing scientific debates on how connectedness to God and specific Church organizations (e.g., Protestant Christian, Catholic Christian, and Hindu communities) matters for psychological well-being and physical health outcomes in diverse cultural contexts.

## Data Availability

The dataset will be available by request from the corresponding author.

## Appendix

See Table 5.

**Table 5:** Demographic characteristics of the sample

Demographic variables	Categories	Frequency	%age
*Gender*			
	Male	42	9.70
	Female	392	90.30
*Racial backgrounds*			
	African American	45	10.40
	American Indian/Alaska Native	2	0.50
	Latino	59	13.60
	Asian	10	2.30
	White	294	67.70
	Mixed race	18	4.10
	Native Hawaiian or other Pacific Islander	1	0.20
	Others	3	0.70
	Prefer not to say	2	0.50
*Religious affiliations*			
	Muslim	2	0.50
	Christian (Protestant, Catholic etc.)	347	80.0
	Jewish	19	4.40
	Hindu	1	0.20
	Buddhist	2	0.50
	Agnostic	38	8.80
	Atheist	7	1.60
	Others	18	4.10
